# Quality-by-Design-Assisted Optimization of Carvacrol Oil-Loaded Niosomal Gel for Anti-Inflammatory Efficacy by Topical Route

**DOI:** 10.3390/gels9050401

**Published:** 2023-05-10

**Authors:** Mohammed Ghazwani, Umme Hani, Aftab Alam, Mohammed H. Alqarni

**Affiliations:** 1 Department of Pharmaceutics, College of Pharmacy, King Khalid University, Abha 61441, Saudi Arabia; uahmed@kku.edu.sa; 2 Department of Pharmacognosy, College of Pharmacy, Prince Sattam Bin Abdulaziz University, Al Kharj 11942, Saudi Arabia; a.alam@psau.edu.sa (A.A.); m.alqarni@psau.edu.sa (M.H.A.)

**Keywords:** carvacrol, essential oil, niosomes, confocal laser scanning microscopy, anti-inflammatory, dermatokinetics, in vitro drug release studies

## Abstract

Niosomes are multilamellar vesicles that effectively transfer active ingredients into the skin’s layers. To improve the active substance’s penetration across the skin, these carriers are frequently utilized as topical drug delivery systems. Essential oils (EOs) have garnered significant interest in the field of research and development owing to their various pharmacological activities, cost-effectiveness, and simple manufacturing techniques. However, these ingredients undergo degradation and oxidation over time, leading to a loss of functionality. Niosome formulations have been developed to deal with these challenges. The main goal of this work was to create a niosomal gel of carvacrol oil (CVC) to improve its penetration into the skin for anti-inflammatory actions and stability. By changing the ratio of drug, cholesterol and surfactant, various formulations of CVC niosomes were formulated using Box Behnken Design (BBD). A thin-film hydration technique using a rotary evaporator was employed for the development of niosomes. Following optimization, the CVC-loaded niosomes had shown: 180.23 nm, 0.265, −31.70 mV, and 90.61% of vesicle size, PDI, zeta potential, and EE%. An in vitro study on drug release discovered the rates of drug release for CVC-Ns and CVC suspension, which were found to be 70.24 ± 1.21 and 32.87 ± 1.03, respectively. The release of CVC from niosomes best fit the Higuchi model, and the Korsmeyer–Peppas model suggests that the release of the drug followed the non-Fickian diffusion. In a dermatokinetic investigation, niosome gel significantly increased CVC transport in the skin layers when compared to CVC–conventional formulation gel (CVC-CFG). Confocal laser scanning microscopy (CLSM) of rat skin exposed to the rhodamine B-loaded niosome formulation showed a deeper penetration of 25.0 µm compared to the hydroalcoholic rhodamine B solution (5.0 µm). Additionally, the CVC-N gel antioxidant activity was higher than that of free CVC. The formulation coded F4 was selected as the optimized formulation and then gelled with carbopol to improve its topical application. Niosomal gel underwent tests for pH determination, spreadability, texture analysis, and CLSM. Our findings imply that the niosomal gel formulations could represent a potential strategy for the topical delivery of CVC in the treatment of inflammatory disease.

## 1. Introduction

Due to their low cost and simple manufacturing processes, the utilization of EO constituents for biomedical applications has attracted global interest [1]. Their numerous qualities, such as viricidal, bactericidal, fungicidal, local anaesthetic, anti-inflammatory, and analgesic properties, have made them widely utilized for centuries. Because they are relatively safer for the skin than synthetically made traditional drugs, EO are often chosen as priority [2]. The components derived from plants still have the potential to cause allergies, but they are used in much lower concentrations than directly applying the oils to the skin, making them a safer option [3]. EOs are mostly made up of volatile substances such as aliphatic, aromatic, terpenoids and phenolic compounds. However, these chemicals could slowly deteriorate and oxidize, which would reduce their functionality [4]. Additionally, the bioavailability, flavour, and colour of the other ingredients may change in response to one another [5]. EOs are substances that, from a physiological standpoint, rapidly evaporate and/or decay even while the temperature is milder than the room temperature. This is because their boiling temperatures are extremely low, and they break down when directly exposed to light or oxygen, in general. Additionally, EO formulations frequently result in skin sensitivity and irritation [5]. The plant species *Thymus vulgaris*, *Origanum vulgare*, *Ocimum gratissimum*, *Carum copticum*, *Oliveria decumbens*, *Trachyspermum ammi*, *Monarda didyma*, *Nigella sativa*, *Origanum onites*, *Origanum syriacum*, *Plectranthus amboinicus*, *Lavandula multifida*, and *Satureja thymbra* are the primary sources of thymol (2-isopropyl-5-methylphenol; C_10_H_14_O), an isomeric form of carvacrol (5-isopropyl-2-methylphenol; C_10_H_14_O) [6,7]. Carvacrol and thymol have similar chemical structures and biological functions, which may indicate that they have related action mechanisms and therapeutic benefits. The CVC possesses a wide range of therapeutic activity, including those of an immunomodulator, an antioxidant, an antiseptic, an anti-diabetic, a cancer prophylactic agent, an anti-inflammatory compound, an anti-spasmodic molecule, and a growth promoter [8,9,10,11,12,13,14,15]. CVC is a promising drug for the treatment of local skin damage [16,17]. However, its pharmaceutical use is severely constrained by its lower bioavailability as a result of its poor water solubility, high volatility, and instability [10]. CVC exhibited anti-inflammatory activity by reducing cyclooxygenase-2 (COX-2) and inhibiting interleukin-1β (IL-1β), and potentially induced interleukin-10 (IL-10) release [18]. The release of IL-10 would result in an anti-inflammatory impact because IL-10 is an anti-inflammatory cytokine that inhibits several cell surface molecules, such as the MHC class II proteins and co-stimulatory molecules [19,20]. Many strategies have been put up to overcome this solubility and bioavailability. One of the flexible approaches involves encasing the EOs in other nanoparticles, such as liposomes [21], niosomes [22], graphene oxide [23], and others. Niosomes can extend drug retention duration, increase solubility, and improve targeted delivery [24].

Niosome is a biocompatible bilayer system based on non-ionic surfactants and cholesterol (as a lipid) [25]. The amphiphilic non-ionic surfactant self-assembles to produce niosomes in aqueous fluids. Niosomes also have several benefits, such as the ability to encapsulate both hydrophilic and hydrophobic components, improved therapeutic performance, long storage times, easy surface modification, biodegradability, and non-immunogenicity [26]. Entrapping EOs in the hydrophobic areas of niosomes can increase their permeability and stability qualities, lowering their potential for skin irritation [27]. These nonionic surfactant vesicles enhance the potential of the entrapped drug when applied topically by modifying the properties of the stratum corneum, facilitating the transdermal transport of trapped substances and decreasing trans-epidermal water loss [28]. Niosomal formulations have been created for a variety of targeted drug delivery purposes throughout the past decade [29]. To produce small-sized niosomes with maximal encapsulation efficiency for any given drug, however, the size and drug encapsulation efficiency of the niosomes must be optimized by altering the formulation (surfactants and lipids) [30,31]. Niosomal formulations of compounds containing CVC oil have been developed for anti-inflammatory and other studies, but a CVC-loaded niosomal formulation has not yet been developed [32,33,34,35,36]. The primary objective of this study was to investigate the potential of CVC for the development of niosomes. Niosome efficacy can be affected by many factors related to formulation and processing. Investigation into these aspects of niosome preparation would thus significantly improve the formulation. The quality by design (QbD) strategy involves the design and development of a product with manufacturing procedures that meet predetermined product criteria [37]. To ensure that selection criteria for desired responses are established for a range of vesicle sizes (<200 nm), polydispersity index (PDI) (<0.3), and entrapment efficiency (>70%) for formulation optimization, response surface methodology (RSM) was employed [38]. For the preparation of niosomes in drug delivery, a variety of nonionic surfactants, including polysorbates, alkyl esters, and alkyl ethers, are usually used. An important factor influencing the physicochemical properties of niosomes, such as bilayer rigidity, vesicle size, stability, and drug release rate, is the type of surfactant used [39]. Tween 80 and Span 80 have undergone testing as drug delivery systems, and specifically, they can be used to create highly stable niosomes [40]. In this formulation of niosomes, tween 80 was used as a surfactant. The stabilization of niosomes that have both hydrophilic and lipophilic domains was accomplished using the surfactant Tween 80. Its longer aliphatic tail causes adsorption on the drug’s surface and allows its hydrophilic portion to potentially reach the aqueous phase. This would completely cover the surface and lessen the interfacial friction between hydrophobic drug vesicles [41]. The larger unsaturated side chain of the tween 80’s oleate component may increase the poorly water-soluble drug CVC’s ability to be enclosed more densely in niosome bilayers [42]. The developed niosomal formulation was incorporated into the gel for better retention time. The vesicle morphology, in vitro drug release, CLSM, dermatokinetic and optimized gel formulation texture analysis was also assessed. It can be anticipated that, the use of quality-by-design approach to optimise the CVC-loaded niosomal gel formulation is a novel approach that can help to ensure the formulation’s quality, safety, and efficacy. Furthermore, using a topical niosomal formulation can improve carvacrol skin penetration and absorption, making it more beneficial for inflammatory skin disorders that require targeted therapy without systemic absorption. This approach may lead to the development of a more efficient and effective treatment for inflammatory skin disorders.

## 2. Results and Discussion

### 2.1. Optimization of Carvacrol-Loaded Niosomes (CVC-Ns)

The formulation optimization of the CVC-Ns was carried out utilizing a Box–Behnken experimental design with three parameters and three levels. Different independent variables’ individual and combined effects on the responses were assessed. Table 1 displays the minimum (−1), average (0), and maximum (+1) levels of the three factors (drug as A, surfactant as B, and cholesterol quantity as C). Table 1 displays the data of the evaluation of the 17 different compositions of CVC-loaded niosomes from the BBD design. 

**Figure 1:**
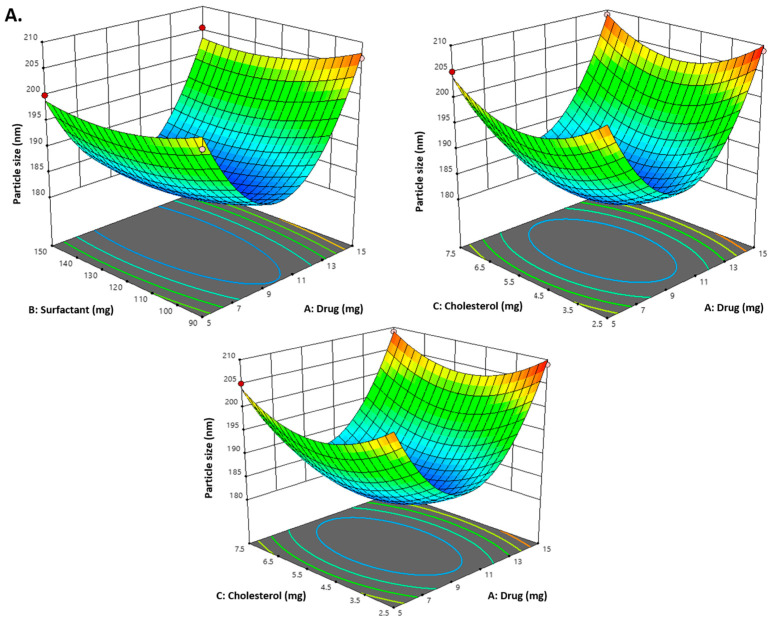

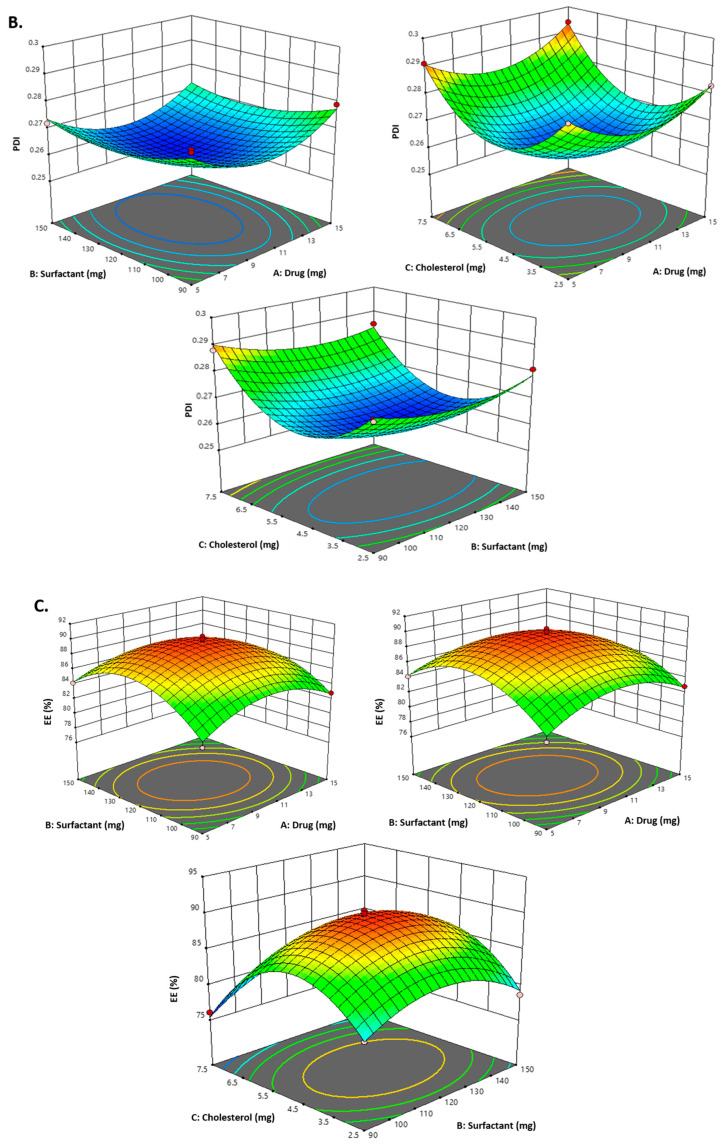
(**A**) Representation of 3D plots of independent variables impacting the dependent variable. Effect of drug conc. (A), surfactant conc. (B) and cholesterol (C) on the PDI (Y_3_). (**B**) Representation of 3D plots of independent variables impacting the dependent variable (PDI). Effect of drug conc. (A), surfactant conc. (B) and cholesterol (C) on entrapment efficiency (%) (Y_2_). (**C**) Representation of 3D plots of independent variables impacting the dependent variable (EE%). Effect of drug conc. (A), surfactant conc. (B) and cholesterol (C) on the vesicle size (Y_1_).

**Figure 2:**
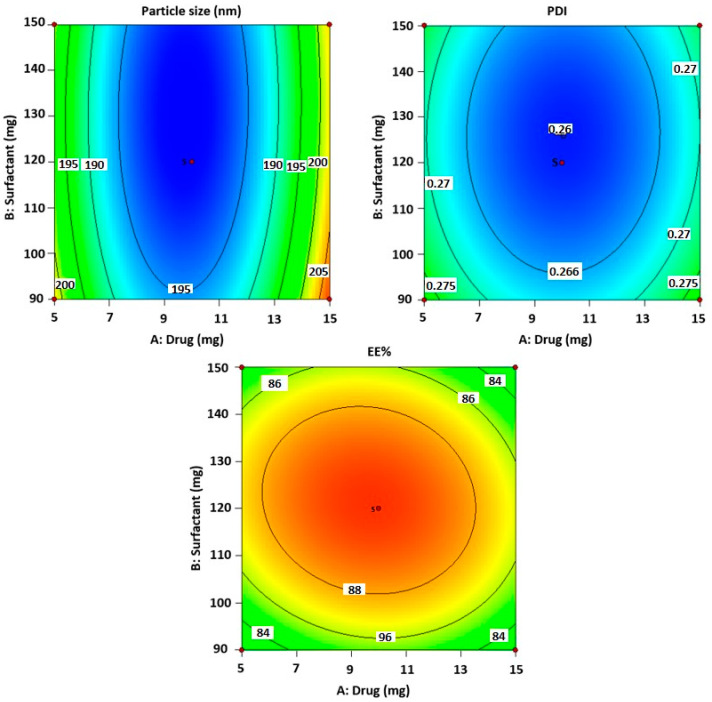
The counterplots for responses for vesicle size, PDI and entrapment efficiency of CVC-Ns.

As indicated in Table 2 and the above-mentioned equation, the CVC-loaded noisome vesicle size ranged from 180.23 to 206.99 nm. Figure 1A and Figure 2 show 3D and contour plots that illustrate the effect of factors on size. The sizes of the niosomes slightly increase when the drug concentration (A) rises. The rise in the concentration of the drug (A) being trapped in the surfactant (B) and cholesterol (C) may be the cause of the size increase [43]. Our research found that the outcomes are consistent with previously published findings: the size increases as the drug concentration in the formulation increases [44]. As per the above equation, surfactant concentration had a negative impact on the size of the vesicles, as increasing the concentration of the surfactant reduces the size of the droplet until the concentration of the surfactant reaches a certain threshold, after which the size of the vesicle increases due to the formation of aggregates by the excess surfactant [45]. Cholesterol, on the other hand, is an important factor responsible for the formation of vesicles: as cholesterol levels rise, vesicle size decreases. This may be because cholesterol above a certain concentration can disrupt the bi-layered structure of vesicular membranes, resulting in drug loss from vesicles [40]. 

According to the equation below, the size’s interaction terms is displayed by the following equation:

Vesicles size = +166.43 + 2.60A − 1.89 B − 0.367 C − 0.380 AB − 1.0723.40 AC + 1.39 BC + 19.61 A^2^ + 2.59 B^2^ + 6.36 C^2^.

All seventeen trials’ PDI values ranged between 0.259 and 0.294. Figure 1B and Figure 2 show 3D and contour plots that illustrate the effect of factors on PDI. The experimental data indicated that the remainder of the independent variable’s surfactant and cholesterol had a negative effect on PDI, indicating that PDI decreased with increasing surfactant concentration until a certain level, as shown in the equation below.

PDI = +0.2614 + 0.001 A − 0.0020 B − 0.0036 C − 0.0003 AB + 0.001 AC − 0.001 BC +0.0101 A^2^ + 0.004 B^2^ + 0.018 C^2^

Cholesterol is an essential factor responsible for vesicle formation: as cholesterol levels rise, vesicle size decreases, resulting in a decrease in PDI. This may be because cholesterol above a certain concentration can disrupt the bi-layered structure of vesicular membranes, resulting in drug loss from vesicles [45].

The following equation displays the EE interaction terms:

EE = +29.68 − 0.3700 A + 0.4338 B − 1.48 C − 0.5750 AB − 0.2540 AC + 0.8075 BC − 2.83 A^2^ − 3.92 B^2^ − 7.60 C^2^

According to the above-mentioned equation, the developed CVC-niosomes had an entrapment efficiency that ranged from 76.08 to 90.41% (Table 2). As per the above equation, the drug concentration increases, and the entrapment efficiency decreases. Figure 1C and Figure 2 show 3D and contour plots that illustrate how factor variables influence the efficiency of encapsulation. The encapsulation effectiveness of the independent factors varied significantly. The effectiveness of the niosome’s encapsulation is favourably impacted by the factor surfactant (B). The encapsulation efficiency increases with a rise in surfactant concentration due to the formation of multiple layers of surfactant molecules around the vesicles and resulting in extra space for incorporating the drug [46]. Moreover, the use of surfactant tween 80 also helped in enhancing the solubilization of the drug molecule by incorporating them [47]. To obtain stable niosomes, cholesterol is the most common additive added to the formulation. It stabilizes bilayers, prevents leakage, and slows the permeation of solutes contained within the aqueous interior of these vesicles. In the present study, the above equation indicates that cholesterol has had a negative effect on entrapment efficiency. The entrapment efficiency of the drug in niosomes was decreased by increasing the cholesterol concentration from 2.5 to 7.5 mg. This may be because cholesterol above a certain concentration can disrupt the bi-layered structure of the vesicular membranes, resulting in drug loss from the vesicles [45].

### 2.2. Design Validation 

To develop the ideal formulation with the lowest globule size, PDI, and highest entrapment efficiency, BBD was used. Table 3 lists the predicted values for all responses and variables derived from the design. Therefore, CVC-N formulations based on the runs, process variables, and responses as shown in Table 2 were created, and the experimental findings attained using the formulations were compared to the expected responses. The fact that the experimental and predicted numbers were nearly closed supports the validity of the optimization process [48]. 

### 2.3. Optimized Composition

The optimized formula obtained from BBD for formulating CVC-loaded niosomes, drug (9.76 mg), surfactant (131.02 mg), and cholesterol (4.62 mg). Optimized formulation was further characterized for various parameters. The chosen formulation for the optimized niosomes was formulation code F4, which contains the drug (mg) (A) 10, surfactant (mg) (B) 120, and cholesterol (mg) (C) 5.

### 2.4. Characterization of CVC-Ns

**Figure 3:**
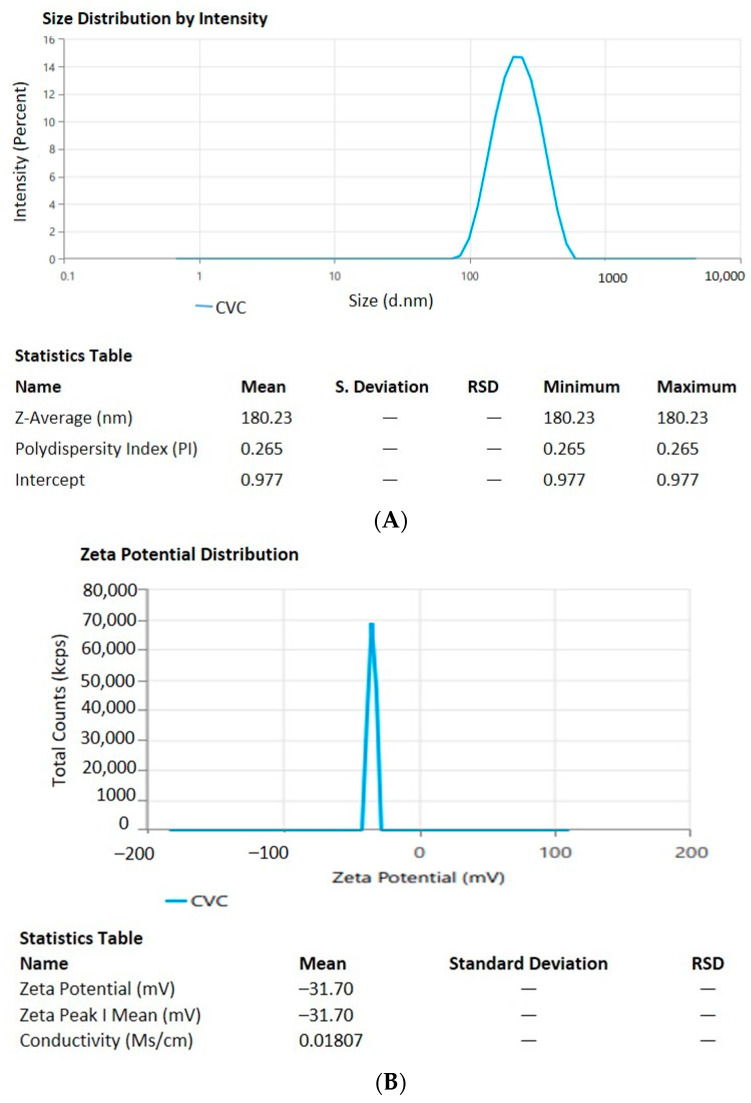
(**A**). Vesicle size and distribution of CVC-loaded niosomes. (**B**). Zeta potential of CVC-loaded niosomes.

For measurements of the vesicle size, Polydispersity Index (PDI), and zeta potential (ZP), the optimal formulation of CVC-Ns was carefully developed based on the characteristics of obtaining the ideal vesicle size and PDI with zeta potential. For the optimized niosomal formulation, the vesicle size distribution (PDI) was 0.265 ± 0.11, and the mean vesicle size was 180.23 ± 1.21 nm. Figure 3A,B, respectively, show the size and zeta potential curve of the optimized formulation. The optimized zeta potential was found to be −31.70 ± 1.11 mV; the zeta potential magnitude gives stability potential of the formulation. All particles in suspension with a large negative or positive zeta potential will resist each other and thus have a low tendency to aggregate [49].

#### Entrapment Efficiency and Drug Loading

The % entrapment efficiency and % drug loading of optimized CVC-Ns was found to be 90.61 ± 2.14% and 60.40 ± 0.99%.

### 2.5. Morphology of CVC-Ns

**Figure 4:**
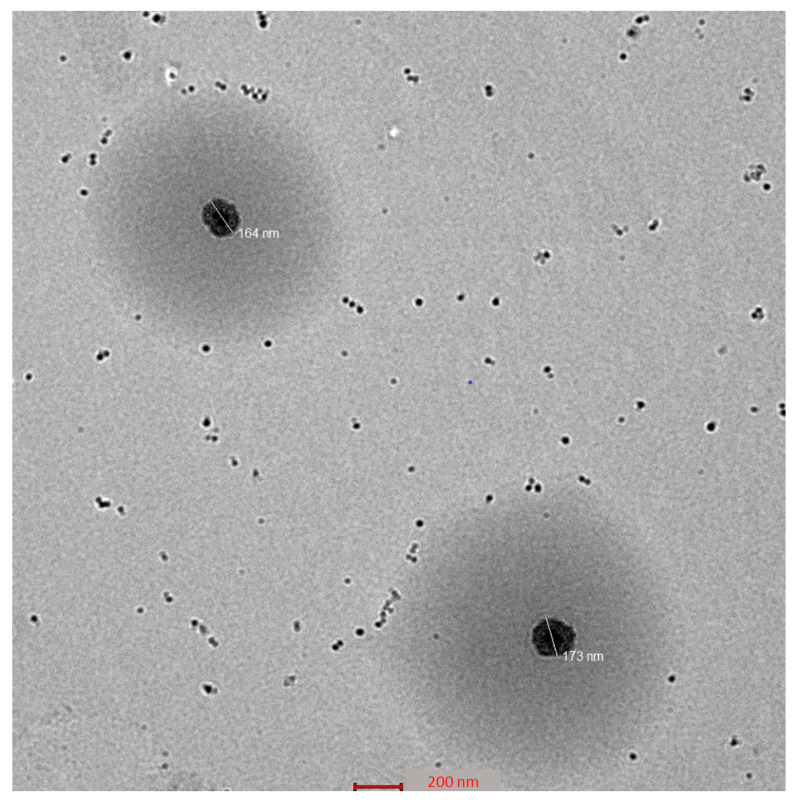
Surface morphology of optimized niosomes.

The optimized CVC-Ns formulation’s TEM picture displays the produced vesicles to be well-defined sealed structures with uniform size distribution and spherical morphologies (Figure 4). The spherical vesicles indicate that CVC is entrapped by niosomes. These vesicles are spherical in shape and homogeneous. The other spots represent the small amount of CVC entrapment in the niosomes [50].

### 2.6. In Vitro Release Study

**Figure 5:**
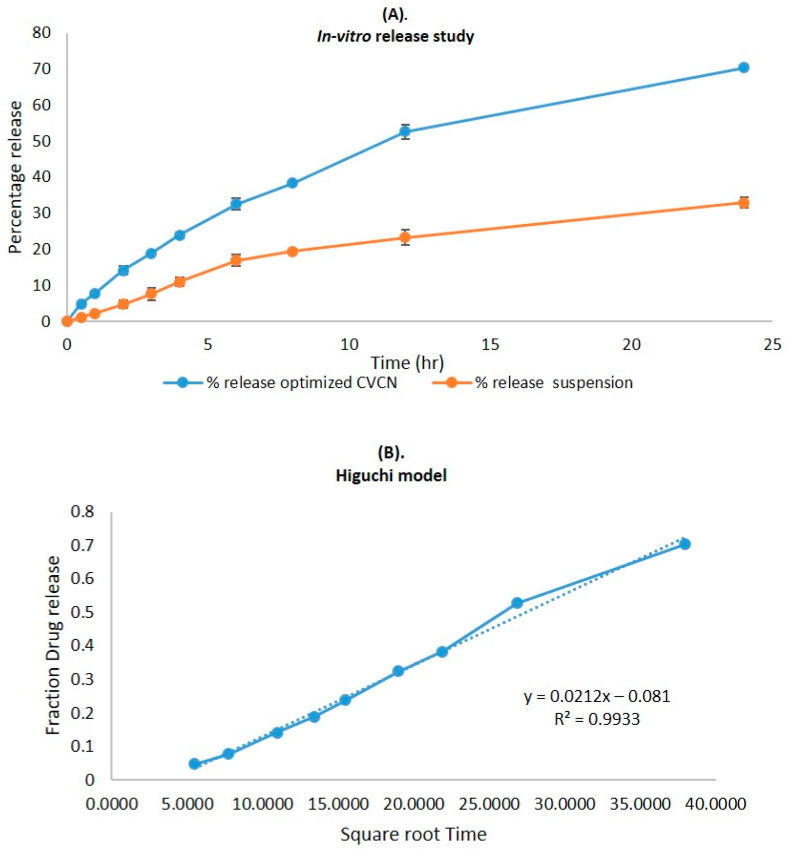

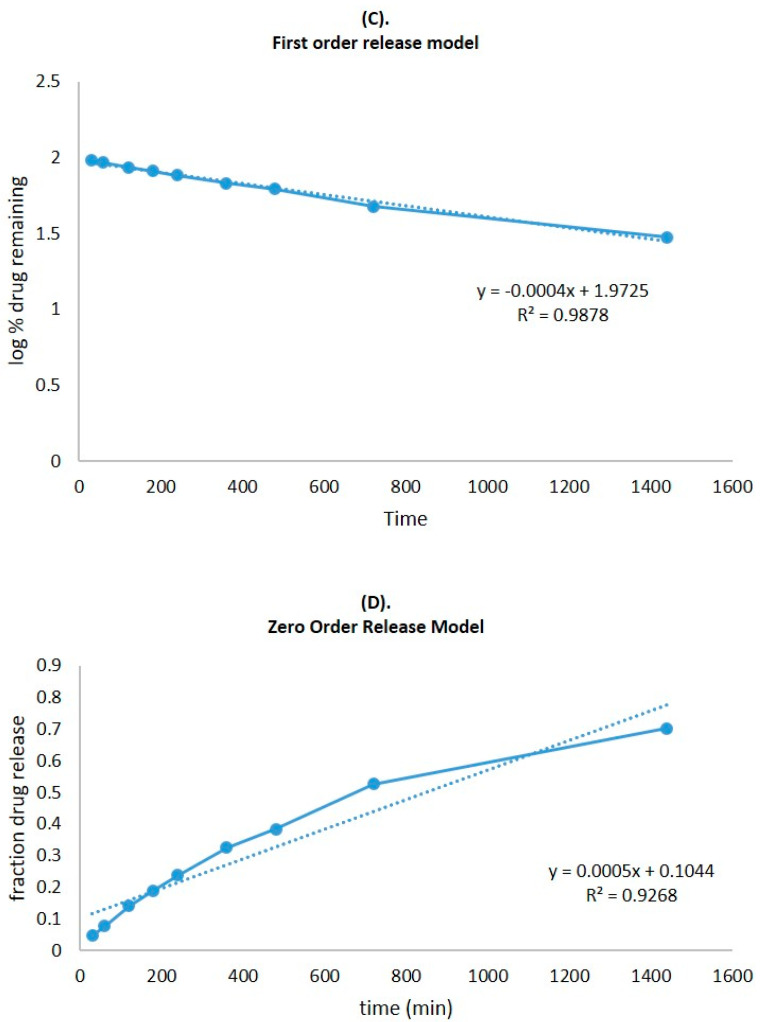

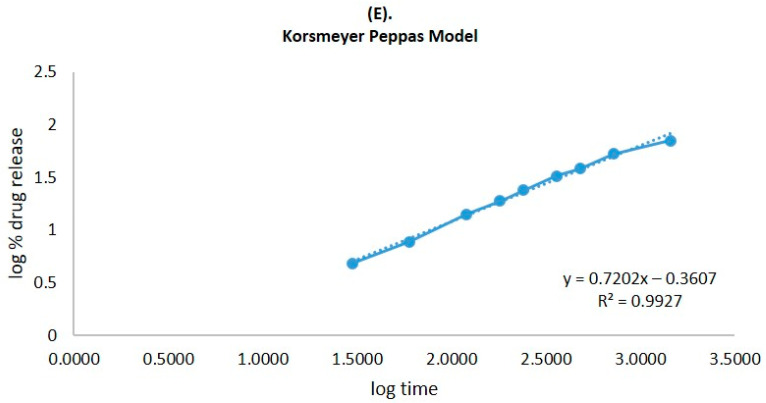
(**A**) Comparison of the optimized CVC-Ns formulation’s in vitro release profile with that of CVC suspension. The investigation was performed in triplicate, and the data are depicted as mean ± SD. (**B**) Higuchi release kinetics of CVC-niosome formulation. (**C**) Release kinetics for a first order and (**D**) release kinetics for zero order of CVC-loaded niosomes. (**E**) Korsmeyer–Peppas model for predicting the release of the drug.

The CVC-loaded optimized niosomal formulation represents a release of CVC through the dialysis bag of 70.24 ± 1.21%, compared to the in vitro CVC release of CVC-suspension, which was found to be 32.87 ± 1.03% (Figure 5A). More than 10–15% of the drug was released in the early two hours, followed by a sustained release for the next 24 h. Initially, the fast release was caused by the rapid release of CVC from the niosome surface, but later, a slower release phase was discovered. The delayed phase in drug release was controlled by diffusion through swollen niosomal bilayers [51]. The results of the in vitro drug release experiment were analysed by applying several mathematical kinetic models to the data. It was discovered that the value of the correlation coefficient (R^2^) for the release of CVC from optimized CVC-loaded niosomes for the Higuchi matrix model had the greatest value (R^2^ = 0.9933) (Figure 5B). First-order (R^2^ = 0.9878) and zero-order (R^2^ = 0.9268) models were found to have the lowest R^2^ values, as shown in Table 3 and Figure 5C,D. Consequently, after obtaining the highest value of the correlation coefficient, the optimized CVC-Ns indicated Higuchi’s model as the best-fit model. The Korsmeyer–Peppas model was used to fit data to analyses of the release mechanism of CVC from optimized CVC-Ns (Figure 5E). The R^2^ value was found to be 0.9927, and the n value was found to be 0.45 < n < 0.89, indicating that the release mechanism of CVC from optimized CVC-N follows non-Fickian diffusion [52].

### 2.7. Analysis of the Gel and Texture of the Optimized CVC-N Gel 

**Figure 6:**
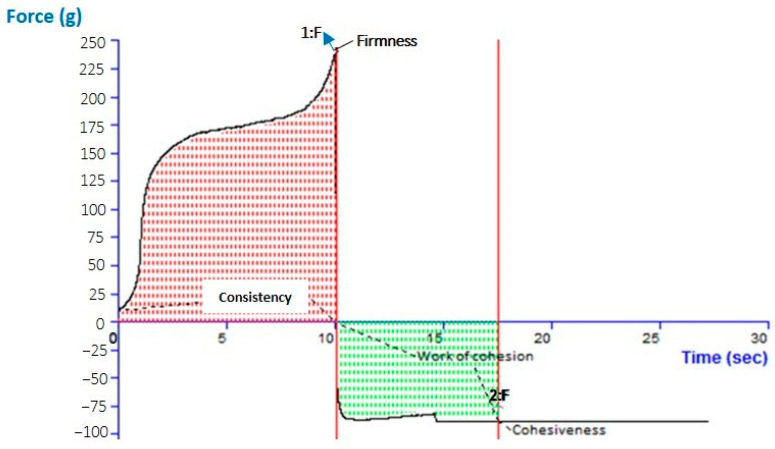
Texture analysis of optimized CVC-N gel.

The pH for the CVC-N gel formulation was 6.01, which is quite close to the skin’s pH [52]. According to data on texture analysis, as shown in Figure 6, the CVC-N gel has the following properties: firmness of 239.09 g, consistency of 1587.00 gm sec, cohesiveness of −89.17 gm, and work of cohesion of −645.47 gm sec, as shown in Table 4 [53]. Peak or maximum force is used to measure firmness; the higher the number, the thicker the sample’s consistency. The sample’s stickiness or cohesiveness is determined by measuring the greatest negative force. The stiffer the sample is, the more negative the number [54]. 

### 2.8. Confocal Laser Scanning Microscopy

**Figure 7:**
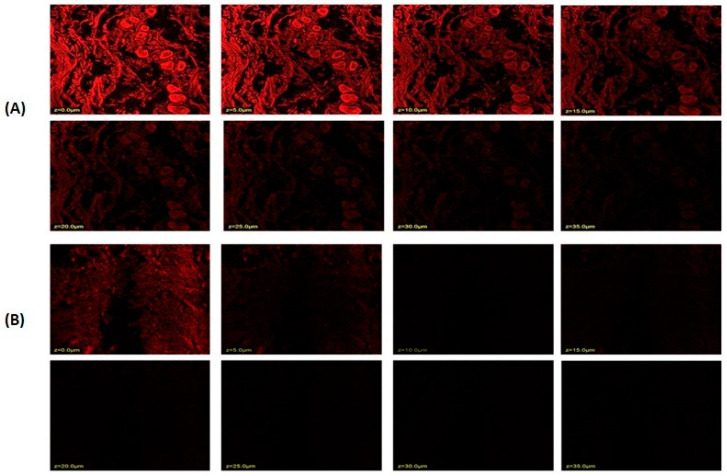
Confocal laser scanning microscopy; (**A**) niosome-formulation-loaded rhodamine B dye; (**B**) hydroalcoholic solution of rhodamine B dye.

The stratum corneum thickness of rat or human skin is reported to be 18 and 17 μm, respectively, and the active site for most of the therapeutic complications associated with the skin lies below the stratum corneum; thus, a drug moiety must penetrate at least 20–200 μm across the skin to exert therapeutic effect [55]. The results showed that niosomal gel loaded with rhodamine B penetrated a deeper layer to a depth of 25.0 μm (Figure 7A). Rhodamine B hydroethanolic solution only exhibited infiltration up to 5.0 μm, suggesting that the dye was only present in the top layers of the rat’s skin (Figure 7B). In contrast, because the fluorescence intensity is greater in the centre of the skin, it is probable that the formulation was maintained in the lower epidermal area of the rat skin. Many skin conditions that affect this lower epidermal skin region require the formulation loaded with rhodamine B to remain in the skin. Thus, it could be said that the created niosomal gel effectively carried rhodamine B dye deeper into the rat’s skin layers.

### 2.9. Dermatokinetic Studies

**Figure 8:**
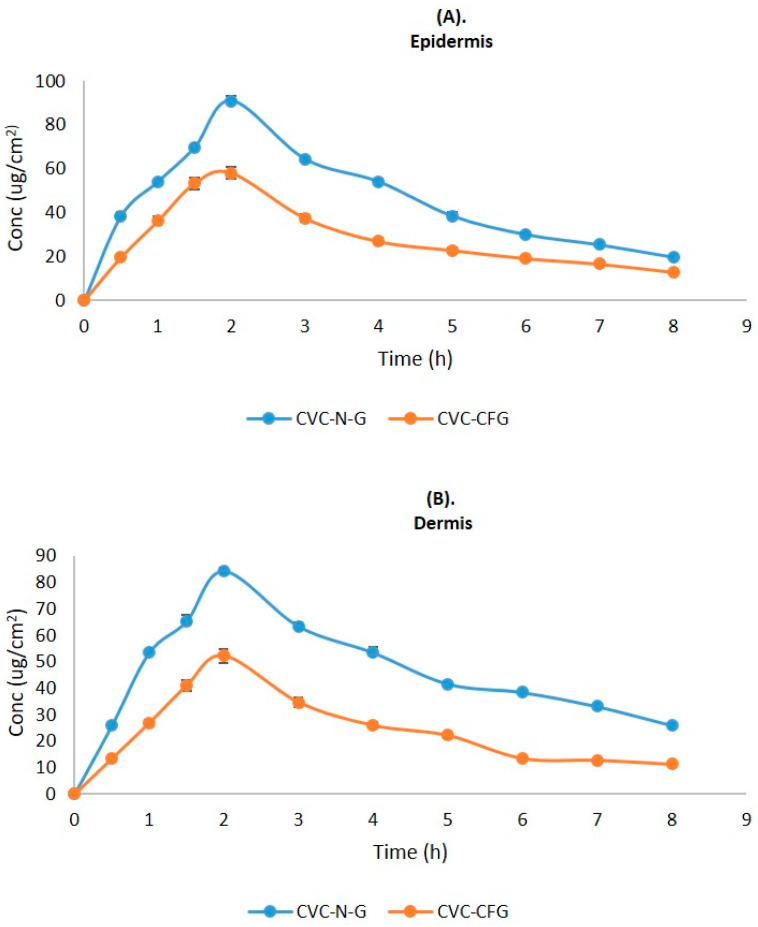
CVC concentration on the (**A**) epidermis and (**B**) dermis of excised rat skin following application to the skin of CVC-N gel and CVC-CF gel.

As a result of the application of CVC-CF gel and CVC-N gel at a predefined time interval, the CVC concentration in the dermis and epidermis of the rat’s skin is shown in Figure 8. Table 5 displays the values of the dermatokinetic parameters. The rat skin that had been exposed to CVC-CF gel displayed C_Skin max_ values of 179.04 ± 0.96 μg/cm^2^ in the epidermis and 160.13 ± 0.64 μg/cm^2^ in the dermis. A C_Skin max_ value of 283.54 ± 1.01 μg/cm^2^ was found in the epidermis, and 262.64 ± 1.12 μg/cm^2^ was found in the dermis of a rat whose skin had been treated with CVCN gel. AUC_0-t_ values of 677.47 ± 0.28 and 572.23 ± 0.31 μg/cm^2^ h, respectively, were seen in the epidermis and dermis of a rat whose skin had been treated with CVC-CF gel. AUC_0-t_ values in the epidermis and dermis of rat skin treated with CVC-N gel, in contrast, were 1135.5 ± 0.64 and 1158.7 ± 1.08 μg/cm^2^ h, respectively. When rat skin was treated with CVC-N gel in contrast to the CVC-CF gel formulation, a greater percentage of CVC was maintained in both rat skin layers. The epidermis and dermis both showed greater C_Skin max_ and AUC_0-t_ values, which demonstrate that the CVC-N gel improved drug absorption in both the epidermal and dermis layers because of its nanosized vesicles’ ease of penetration into the skin’s lipid layers, enhancing its therapeutic efficacy in the treatment of inflammatory diseases [56].

### 2.10. Ferric-Reducing Antioxidant Power (FRAP)

The analysis indicated that CVC has a significant amount of antioxidant capacity and that the agent is effective at scavenging free radicals, and preventing the oxidation of antioxidant activity was found to be 60.14 ± 1.11% in free CVC, 88.41 ± 2.32% in an ascorbic acid solution, and 71.24 ± 3.31% in a CVC-N-optimized formulation. As the results indicate, CVC-Ns have been shown to have a significant antioxidant effect.

### 2.11. Stability Studies

The data of experiments into short-term accelerated stability were evaluated for six months. Physical appearance, shape, vesicle size, PDI, zeta potential, colour appearance, phase separation, clarity, homogeneity, pH, and drug content were detected (Table 6 and Table 7). This study thus validates the notion that niosomes are durable over long periods of storage.

## 3. Conclusions

Niosomes were effectively developed using thin film hydration. Using BBD, the niosomal formulation was optimized by taking independent and dependent factors. The optimized formulation achieved the highest amount of CVC entrapping, suggesting that it was the most ideal among all formulations, i.e., batch code F4. The optimized CVC-loaded niosomes had reduced PDI values and were in the colloidal size range, which indicated that the formulations were homogeneous. The TEM images demonstrated the spherical shapes of the vesicles, providing evidence of an entrapped CVC. According to in vitro drug release studies, the amount of CVC released from CVC-loaded niosomes was two times higher than it was from CVC suspension solution, demonstrating improved drug release from the niosome formulation due to nanosized vesicles. A CLSM study on rat skin has provided insight into the penetration of rhodamine-loaded B hydroalcoholic and rhodamine-loaded niosome formulations, and the results showed that rhodamine-B-loaded niosomal formulations penetrated the skin much more effectively than hydroalcoholic solutions of the rhodamine B dye, indicating that niosomes have improved in vivo prospects for anti-inflammatory treatment. Compared to CVC-CFG, dermatokinetic studies show that a higher concentration of CVC-NG reaches the epidermis and dermis (Cskin max and AUC0-t) due to the nanosized vesicles providing a means to cross the stratum corneum to have a profound effect. The antioxidant studies revealed that the niosome formulation has a greater potential for antioxidants than the pure drug, showing a capability to reduce the level of free radicals and reactive oxygen stress and hence to reduce inflammation. Moreover, the results of this experiment confirmed that the developed, optimized niosome formulation is an effective means of delivering CVC drugs topically and can therefore be used to treat anti-inflammatories more successfully. However, the actual skin penetration of carvacrol may vary depending on the formulation and the distinctive characteristics of the skin. While carvacrol is usually regarded as safe, the safety profile of carvacrol-containing niosomes for topical distribution has not been fully established, and additional research may be required to determine their possible toxicity and side effects and to validate the in vitro and skin permeation results by using appropriate animal models in preclinical studies.

## 4. Materials and Methods

### 4.1. Materials

Carvacrol oil, polyethylene glycol 400 (PEG), triethanolamine rhodamine-123, Ascorbic acid and potassium ferricyanide were procured from Sigma-Aldrich (St. Louis, MI, USA). Cholesterol and tween 80 were provided by S D Fine Chemicals Ltd. (Mumbai, India). Methanol and chloroform were provided by Merck Mumbai, India. B.S. Goodrich in Pleveland generously provided carbopol-934 as a gift sample. Other agents used in the experiment such as disodium hydrogen phosphate, potassium dihydrogen phosphate, and sodium chloride of analytical grade were provided by S D Fine Chemicals Limited, Mumbai, India.

### 4.2. Method

#### 4.2.1. Preparation of CVC-Loaded Niosomes (CVC-Ns)

Niosomes loaded with CVC (CVC-Ns) were made using the thin-layer hydration method. Briefly, the drug (CVC), cholesterol, and surfactant were dissolved in a round-bottomed flask using a chloroform-to-methanol ratio of 2:1. The organic phase was then evaporated in a rotary evaporator (120 rpm, 60 °C, 1 h) to generate a thin layer. Phosphate-buffered saline solution (PBS pH—6.8, 10 mL) was then used to rehydrate the desiccated thin film at room temperature (30 °C) for one hour (200 rpm). CVC-loaded niosomes were then sonicated for 2 min by a probe sonicator. The samples were preserved in a refrigerator at 4 °C for further investigation [57].

#### 4.2.2. CVC-Loaded Niosome Optimization through Experimental Design

The Box–Behnken design (BBD) of the Design-Expert software (Stat-Ease Inc.’s, Design Expert^®^, Version 13) was used to examine the relationships between the three selected input variables (drug, surfactant, cholesterol). The output variables studied were the vesicle size (Y_1_), polydispersity index (PDI, Y_2_) and entrapment efficiency (Y_3_). The input variables were chosen, at their three levels, i.e., their minimum, average, and maximum values, which are shown in Table 8 to explore the final optimal formulation [58].

### 4.3. Characterization of CVC-Ns

#### 4.3.1. Determination of Globule Size and Zeta Potential 

To determine the zeta potential and vesicle size distribution, the dynamic light scattering (DLS) method was utilized. Before analysis, samples were 100-fold diluted in double-distilled water, and impurities were removed using 0.45 m membrane filters. The Malvern particle size analyser equipment (Malvern Instrument Ltd., Malvern, UK) was utilized to determine the zeta potential and to quantify the vesicle size [59].

#### 4.3.2. Measurement of % Entrapment Efficiency and % Drug Loading 

The drug loading and entrapment efficiency of CVC-loaded niosome vesicles were determined by ultra-centrifuging (Beckman Coulter India Pvt. Ltd., Mumbai, India) formulations for one hour at 15,000 r/min and 4 °C. The upper layer of liquid (supernatant) was withdrawn using a pipette, diluted, and filtered (0.25 µm), and the CVC concentration was measured employing UV spectroscopy (UV 1601, Shimadzu, Nagoya, Japan) at 280 nm [60]. The experiment was performed in triplicate. For evaluating the drug-loading capability of the niosomes, drug entrapment efficiency is a crucial factor. This parameter depends on the developmental methodology, the physicochemical features of the drug, and the formulation factors [61].

The equation was used to obtain the entrapment efficiency (EE%) and drug loading (%) [62]:

$$\mathrm{Encapsulation}\;\mathrm{efficiency}\;\left(\%\right)=\frac{\left(\mathrm{total}\;\mathrm{amount}\;-\mathrm{free}\;\mathrm{amount}\right)}{\mathrm{total}\;\mathrm{amount}\;}\times 100$$

$$\mathrm{Drug}\;\mathrm{loading}\;\left(\%\right)=\frac{\mathrm{amount}\;\mathrm{of}\;\mathrm{drug}\;\mathrm{entraped}}{\mathrm{total}\;\mathrm{amount}\;\mathrm{of}\;\mathrm{drug}\;\mathrm{and}\;\mathrm{lipid}}\times 100$$

### 4.4. Morphological Studies 

Transmission electron microscopy (TEM) analysis was carried out on a JEOL-2000 Ex II TEM (Akishima, Japan) to morphologically characterise the niosomes. The excess sample was taken off the carbon-coated copper grid using filter paper after a drop of the niosomal formulation was applied to it. The carbon-coated copper grid was then applied with a drop of 2% (*w*/*v*) PTA (phosphotungstic acid solution) and kept undisturbed for 2 min. The excess of the staining agent was removed with the help of a filter paper, and the sample was air-dried before being examined on the transmission electron microscope [63].

### 4.5. Formulation of CVC Niosomal Gel (CVCNG)

Niosomal dispersions have a very low viscosity, which makes them unsuitable for topical use; therefore, gelling was performed. Carbopol 934 was spread out by slowly adding distilled water and letting it sit in the dark so that it could fully swell. The dispersion was further made neutral by adding triethanolamine drop by drop to achieve the intended result of a viscous transparent gel. Finally, the optimized CVC-N formulation was added slowly to the gel with constant agitation to create CVC-NG [64]. 

### 4.6. In Vitro Drug Release Study

The optimized CVC-N formulation and carvacrol suspension (drug (10 mg)) was dissolved in methanol and the volume was made up to 10 mL with adequate solvent. The drug release investigation was conducted using the cellulose dialysis membrane bag (molecular weight cut off–12,000 kDa). The dialysis bag was filled with both of the prepared formulations (2 mL of each containing 2 mg of the drug), and the ends were knotted. As part of the experiment, the dialysis bag was submerged in 100 mL of the phosphate buffer (pH 6.8; medium). The release media’s temperature was maintained at 37 ± 2 °C throughout the investigation, which was conducted with constant stirring. The sample (1 mL) was taken at 0, 0.5, 1, 2, 4, 6, 8, 12 and 24 h, and precisely the right amount of fresh medium was added to keep the sink conditions. The in vitro drug release study was conducted for 24 h to determine the drug release pattern, the mechanism it follows and how long it remains in the target area. The obtained samples were filtered (0.22 μm) and further diluted to determine the content of the drug by utilizing a UV spectrophotometer at 280 nm. To evaluate the release mechanism, the drug release data were fitted to various release kinetic models, including zero-order, first-order, Higuchi, and Korsmeyer–Peppas models [65]. 

### 4.7. Characterization of Gel

#### Evaluation of pH and Texture of CVC-N Gel

A digital pH meter electrode (Mettler Toledo, Chiyoda, Japan) was utilized to directly measure the pH of the CVC-N gel. TA.XT Plus Texture analyser (Stable Micro Systems Ltd., Surrey, UK) was used to determine the gel’s texture. The texture analysis curve produced at the end of the trials was used to identify parameters including hardness, consistency, cohesiveness, and gel viscosity index [66].

### 4.8. Spreadability Studies

The parallel plate method was used to determine the niosomal gel spreadability. In this procedure, 500 mg of the formulated gel was added to a circle diameter of 1 cm, atop a glass plate that had been pre-marked and another glass plate had been placed on top. Five minutes were given for a weight of 500 gm to lay on the top plate of the glass. It was seen that the gel spreading caused the diameter to expand [67]. 

### 4.9. Permeation Depth Study by Loading Rhodamine B Dye

Franz diffusion cell was utilized in the confocal studies to evaluate the infiltration in the skin by rhodamine-B-dye-loaded niosomal formulation and standard solution of rhodamine B dye. A 1 cm^2^ sample of rat skin was cut from the abdomen, and the hairs were removed using purified water and a phosphate-buffered saline solution; the rat skin was meticulously washed and rinsed until it was completely clean. The stratum corneum was then facing up and the dermis was facing down when the skin was then put on the diffusion cell. The niosome formulation was mixed with rhodamine B, while the standard was hydroalcoholic rhodamine B solution. Both were put into the donor compartment and kept at a temperature of 32 ± 0.5 °C for six hours. Additionally, slides with the stratum corneum pointing upward were examined using a confocal microscope and an optical excitation of 488 nm. Above 560 nm, both the argon laser beam and the fluorescence emission could be seen. Confocal laser scanning microscopy (CLSM) was used to compare the penetration and distribution of improved gel formulation with traditional gel (TCS SP5II; Leica Micro System Ltd., Wetzlar, Germany) [68].

### 4.10. Dermatokinetics 

The dermatokinetic analysis was performed to determine the quantity of drug in different layers of excised rat skin after applying the CVC-NG formulation mounted on a diffusion cell apparatus. The research was carried out following the protocol described in the CLSM study. In this case, the Franz diffusion cell skin samples were obtained for this study at 0, 1, 2, 4, and 8 h. The skin was then washed with normal saline and kept at 60 ± 0.5 °C for 2–3 min. The skin layers (epidermis and dermis) were separated using forceps and sliced into tiny fragments before being soaked in methanol for 24 h to extract the drug contained in the layers. The methanolic extracts of the drug were further filtered (0.22 µm), and the drug concentration was determined using a UV spectrophotometer. All the measurements were performed in triplicate [69].

### 4.11. Ferric-Reducing Antioxidant Power (FRAP)

In brief, 2.5 mL of 0.2 M sodium phosphate buffer (pH 6.6) and 2.5 mL of 1% (*w*/*v*) potassium ferricyanide solution were combined with 1 mL of sample solution (10 µg/mL) in methanol. Employing a method similar to Oyaizu’s with a few minor alterations, the reduction power (antioxidant) of CVC in the niosomal formulation was determined. The mixture was then incubated for 20 min at 50 °C before being treated with 2.5 mL of 10% trichloroacetic acid. Then, 2.5 mL from the top layer of the mixture, 2.5 mL of distilled water, and 0.5 mL of FeCl_3_ (0.1%) were added after the mixture had been centrifuged for 10 min at 3000 rpm. The amount of ascorbic acid that was utilized as the standard was 10 µg/mL, and a spectrophotometer was used to measure the absorbance at 700 nm. Each test was performed in triplicate [70].

### 4.12. Stability Studies 

Optimized CVC-N and CVC-N gel formulations underwent short-term accelerated stability evaluation in line with the International Conference on Harmonization (ICH) Q1 A R2 guidelines. Lyophilized niosomes (CVC-Ns) were placed in an Eppendorf, sealed, and maintained in a stability chamber at 25 ± 2 °C and RH 60 ± 5% at 4 ± 2 °C. The formulation along with the gel was then assessed for changes in physical appearance, shape, vesicle size, PDI, zeta potential, clarity, homogeneity, pH and phase separation for six months [71].

## Figures and Tables

**Table 1:** Responses observed in BBD for development and optimization of CVC-Niosome formulations with a summary of parameters for responses (Y_1_, Y_2_, Y_3_) by Design Expert Software.

Formulations	Independent Variables			Dependent Variables		
	A	B	C	Y_1_	Y_2_	Y_3_
1	5	120	7.5	205.05	0.291	78.49
2	10	120	5	180.98	0.259	88.97
3	10	150	7.5	186.98	0.285	77.95
4	10	120	5	180.23	0.265	90.41
5	10	120	5	181.07	0.261	89.48
6	10	150	2.5	185.89	0.281	78.62
7	15	120	7.5	208.95	0.294	76.38
8	5	120	2.5	204.62	0.286	81.64
9	15	90	5	206.99	0.279	82.84
10	15	120	2.5	208.95	0.283	80.51
11	15	120	5	181.28	0.262	89.55
12	5	150	5	200.01	0.272	84.18
13	10	90	2.5	195.54	0.279	79.98
14	5	90	5	199.94	0.279	81.55
15	10	120	5	181.08	0.26	90.01
16	10	90	7.5	191.08	0.288	76.08
17	15	150	5	205.54	0.271	83.17

A, drug (mg); B, surfactant (mg); C, cholesterol (mg); Y_1_, vesicle size (nm); Y_2_, PDI; Y_3_, EE (%).

**Table 2:** Results of regression analysis of Responses Y_1_, Y_2_ and Y_3_.

Quadratic Model	R^2^	Adjusted R^2^	Predicted R^2^	SD	%CV	*p*-Value
Response (Y_1_)	0.9882	0.9731	0.8162	1.85	0.9395	<0.0001
Response (Y_2_)	0.9777	0.9490	0.7883	0.0026	0.7818	<0.0001
Response (Y_3_)	0.9890	0.9749	0.8673	0.7946	0.9582	<0.0001

**Table 3:** In vitro drug release kinetics of CVC-loaded niosomes.

Kinetic Models	*X*-Axis	*Y*-Axis	CVC-Ns (R^2^)
Zero-order	Fraction of drug released	Time	0.9268
First order	Log % drug remaining	Time	0.9878
Higuchi matrix	Fraction of drug release	Square root time	0.9933
Korsmeyer–Peppas	Log fraction of drug released	Log time	0.9927

**Table 4:** Physiochemical characterization of CVC-based niosomal gel (CVCNG).

Homogeneity	Appearance	Washability	Separation of Phase	Odour
Homogeneous	Translucent	Yes	No	odourless
**Colour**	**Content of Drug (%)**	**pH**	**Spreadability (gm. cm/sec)**	
off-white	90.11 ± 0.98	6.01 ± 1.01	18.27 ± 1.24	
**Cohesiveness (gm)**	**Consistency (gm.Sec)**	**Firmness (gm)**	**Work of cohesion g, sec**	
−89.17	1587.00	239.09	−645.47	

**Table 5:** Dermatokinetic parameters (mean ± SD) of CVC-N gel and CVC-CF gel.

Dermatokinetics Parameters	CVC-N Gel		CVC-CF Gel	
	Epidermis Mean ± SD	Dermis Mean ± SD	Epidermis Mean ± SD	Dermis Mean ± SD
T_skin max_ (h)	2	2	2	2
C_skin max_ (μg/cm^2^)	283.54 ± 1.01	262.64 ± 1.12	179.04 ± 0.96	160.13 ± 0.64
AUC_0-8_ (μg/cm^2^ h)	1135.5 ± 0.64	1158 ± 1.08	677.47 ± 0.28	572.23 ± 0.31
Ke (h^−1^)	0.171 ± 1.11	0.119 ± 0.91	0.146 ± 0.47	0.119 ± 0.78

**Table 6:** Evaluation of the CVC-loaded optimized niosome (CVC-N) formulation’s short-term accelerated stability.

Evaluation Parameters	Initial	1 Month		3 Months		6 Months	
		4 ± 2 °C	25 ± 2 °C/ 60 ± 5% RH	4 ± 2 °C	25 ± 2 °C/ 60 ± 5% RH	4 ± 2 °C	25 ± 2 °C/ 60 ± 5% RH
Appearance	+++	+++	+++	++	++	++	++
Phase separation	NO PHASE SEPARATION						
Shape	Spherical in shape						
PDI	0.259	0.259	0.264	0.271	0.277	0.280	0.284
Vesicle size (nm)	180.23	180.23	180.99	181.11	182.14	183.09	183.21
Zeta potential (mV)	−31.70	−31.70	−32.01	−32.45	−33.41	−33.39	−33.34

++ good, +++ excellent.

**Table 7:** Short-term accelerated stability evaluation of CVC-loaded optimized niosomal gel formulation.

Evaluation Parameters	Initial	1 Month		3 Months	6 Months		
		4 ± 2 °C	25 ± 2 °C/60 ± 5% RH	4 ± 2 °C	25 ± 2 °C/60 ± 5% RH	4 ± 2 °C	25 ± 2 °C/60 ± 5% RH
Colour	Slightly off white						
Appearance	Translucent						
Phase Separation	NO PHASE SEPARATION						
Clarity	√	√	√	√	√	√	√
pH	6.01	6.01	6.29	6.31	6.39	6.55	6.67
Homogeneity	***	***	**	***	**	***	*
Washability	Washable						
Odour	NO						

* satisfactory, ** good, *** excellent.

**Table 8:** Factors and responses employed in BBD to create CVC-Ns.

Factor	The Level Used, Actual Coded		
Factors	Low (−1)	Medium (0)	High (+1)
A = Drug (mg)	05	10	15
B = Surfactant (mg)	90	120	150
C = Cholesterol (mg)	2.5	05	7.5
**Responses**	**Aim**		
Y_1_ = globule size (nm)	<200 nm		
Y_2_ = PDI	<0.3		
Y_3_ = Entrapment Efficiency (%)	>70%		

A, drug (mg); B, surfactant (mg); C, cholesterol (mg); Y_1_, Vesicle size (nm); Y_2_, PDI; Y_3_, EE (%); BBD, Box–Behnken experimental design; Ns, Niosomes; CVC, carvacrol.

## Data Availability

Not applicable.
